# The impact of religious fasting on human health

**DOI:** 10.1186/1475-2891-9-57

**Published:** 2010-11-22

**Authors:** John F Trepanowski, Richard J Bloomer

**Affiliations:** 1 Cardiorespiratory/Metabolic Laboratory, The University of Memphis, Memphis, TN 38152, USA

## Abstract

The past two decades have seen a rise in the number of investigations examining the health-related effects of religiously motivated fasts. Islamic Ramadan is a 28 - 30 day fast in which food and drink are prohibited during the daylight hours. The majority of health-specific findings related to Ramadan fasting are mixed. The likely causes for these heterogeneous findings are the differences between studies in the following: 1) the amount of daily fasting time; 2) the percentage of subjects who smoke, take oral medications, and/or receive intravenous fluids; and 3) the subjects' typical food choices and eating habits. Greek Orthodox Christians fast for a total of 180 - 200 days each year, and their main fasting periods are the Nativity Fast (40 days prior to Christmas), Lent (48 days prior to Easter), and the Assumption (15 days in August). The fasting periods are more similar than dissimilar, and they can each be described as a variant of vegetarianism. Some of the more favorable effects of these fasts include the lowering of body mass, total cholesterol, LDL-C, and the LDL-C/HDL-C ratio. The Biblical-based Daniel Fast prohibits the consumption of animal products, refined carbohydrates, food additives, preservatives, sweeteners, flavorings, caffeine, and alcohol. It is most commonly partaken for 21 days, although fasts of 10 and 40 days have been observed. Our initial investigation of the Daniel Fast noted favorable effects on several health-related outcomes, including: blood pressure, blood lipids, insulin sensitivity, and biomarkers of oxidative stress. This review summarizes the health-specific effects of these fasts and provides suggestions for future research.

## Introduction

Fasting is defined as a partial or total abstention from all foods, or a select abstention from prohibited foods. As a potential non-pharmacological intervention for improving health and increasing longevity, fasting has been the subject of numerous scientific investigations. The three most commonly studied fasts are caloric restriction (CR), alternate-day fasting (ADF), and dietary restriction (DR). A summary of the main findings is presented below.

CR is the reduction of kilocalorie (kcal) intake by a certain percentage (typically 20 - 40%) of *ad libitum *consumption. CR has been demonstrated to improve health and increase longevity in a diverse group of species, including: dog, fruit fly, nematode, rodent, rotifer, spider, non-human primate, and zebrafish [1]. Additionally, CR appears to delay the onset of the following diseases: autoimmune diseases, atherosclerosis, cardiomyopathies, cancer, diabetes, renal diseases, neurodegenerative diseases, and respiratory diseases [2,3]. Regarding cardiovascular health, the following changes have been noted following a CR regimen: decreases in resting heart rate (HR) and blood pressure (BP); increases in HR variability; and improvements in left ventricular function, post-exercise recovery of both HR and BP, and flow-mediated vasodilation [4]. Regarding glucoregulatory health, CR has been shown to decrease fasting glucose and insulin levels, increase insulin sensitivity, decrease body fat percentage, and lower the incidence of diabetes [5,6].

ADF consists of alternating 24-hour periods: during the "feast period," fasters may consume food *ad libitum*; during the "fast period," food consumption is restricted or halted altogether. Water is allowed *ad libitum *during all times. Animal ADF trials have reported extended lifespan [1] as well as the retardation or altogether prevention of the development of many morbidities, including: cardiovascular disease, kidney disease, cancers, and diabetes [4,7]. ADF has been noted to elicit the following beneficial changes in cardiovascular health: decreased HR and BP, increased HR variability, and attenuated post-infarct chronic heart failure [4,8,9]. Regarding glucoregulatory health, ADF may have gender-specific effects. For example, ADF improved insulin sensitivity in men but had no effect on this variable in women [7]. Furthermore, glucose tolerance was unchanged in men partaking in an ADF regimen, but women partaking in the same regimen experienced impaired glucose tolerance [7].

DR is a reduction of one or more components of dietary intake (typically macronutrients) with minimal to no reduction in total kcal intake. Research suggests that neither carbohydrate restriction nor lipid restriction extend life [10]. On the other hand, protein restriction increases maximum lifespan by roughly 20% [11], and this extension of life may be solely due to the reduction of the amino acid methionine [12]. These findings are in reference to animals, so further work involving human test subjects is necessary before firm conclusions can be made.

While religious fasts are partaken primarily for spiritual purposes, they also have the potential to greatly affect one's physical health. Accordingly, the health effects of religious fasting have recently been the subject of scientific inquiry, with most of the research being performed in the last two decades. The following religious fasting periods are featured in this review: 1) Islamic Ramadan; 2) the three principal fasting periods of Greek Orthodox Christianity (Nativity, Lent, and the Assumption); and 3) the Biblical-based Daniel Fast. The reason for the inclusion of these specific forms of religious fasts and the exclusion of others is that, to our knowledge, these are the only fasts about which scholarly research has been performed that explicitly detailed the subjects' dietary intake and health-related outcomes. Specifically, this review will examine the effects of these fasts on energy and nutrient intake, anthropometry, hematometry, blood pressure, and other health-related biomarkers. This paper concludes with a summarization of the findings and suggestions for future research.

### Ramadan

Each year, millions of Muslims refrain from eating or drinking from sunrise (Sahur) to sunset (Iftar) during the holy month of Ramadan, which lasts between 28 and 30 days. Thus, Ramadan fasting is similar to ADF, because both fasts incorporate feast periods and fast periods. The feast periods and fast periods of Ramadan fasting are each 12 hours in length on average [13], which amounts to half of the 24-hour length for both the feast periods and fast periods of ADF. Another important difference between the two forms of fasting is that fluid intake is forbidden during the fast periods of Ramadan, whereas it is permitted at all times under an ADF protocol.

The common dietary practice of Ramadan fasting is to consume one large meal after sunset and one lighter meal before dawn [14], but some Muslims consume an additional meal before sleeping [15]. Muslims consume a greater variety of foods during Ramadan compared with the rest of the year [16]. Also, sugary foods and drinks are consumed more frequently during Ramadan [17].

Upon reaching puberty, all healthy Muslims are required to partake in the fast. Individuals who are sick, traveling, pregnant, breast-feeding, menstruating, or debilitated are exempt from fasting [18,19]. However, many Muslims who are eligible for exemption choose to fast nonetheless [20].

Due to the fact that the Islamic calendar (Hijra) is a lunar calendar, the first day of Ramadan advances 11 days each year in relation to the Gregorian calendar. Consequently, Ramadan falls on different parts of the seasonal year over a 33-year cycle. This seasonal shift dramatically impacts the amount of daily fasting time that occurs in any given location. Moreover, a location's latitudinal distance from the equator also substantially impacts daily fasting time. While the average fast period during Ramadan is 12 hours in length [13], it can be as long as 22 hours in polar regions during summertime [21]. Fortunately, Muslims living in such regions are permitted to adopt the fast period of either Mecca or the nearest temperate location [21].

The variability in daily fasting time is one of several confounding variables that influence the effect of Ramadan fasting on health-related biomarkers. Other confounding variables include smoking status, medication, diet, and cultural habits [22-24]. Smoking is forbidden during the daylight hours of Ramadan; therefore, a subject population containing a large percentage of smokers could conceivably experience changes in health-related biomarkers simply by virtue of smoking less. This latter point similarly applies to a subject population containing a large percentage of people taking oral medications and/or intravenous fluids, as their usage is also disallowed during the daylight hours of Ramadan [22]. Regarding diet, energy intake during Ramadan has been reported to increase in Saudi Muslims and decrease in Indian Muslims; these discrepant findings are believed to be due to the differences in food choices between the groups [24].

Few definitive conclusions can currently be made regarding the effects of Ramadan fasting on human health, because the collective body of research has noted mostly heterogeneous findings regarding both dietary intake and health-related outcomes. The above-listed confounding variables in studies of Ramadan fasting - particularly the dietary norms of the subjects - are likely the main causes of these heterogeneous findings.

As mentioned above, the majority of findings related to energy and macronutrient intake during Ramadan fasting are mixed. A summary of available findings pertaining to daily energy intake as well as the percentage intake of carbohydrate, protein, and fat is presented in Table 1. Similarly heterogeneous findings exist regarding both saturated fat consumption and monounsaturated fat consumption during Ramadan. Regarding saturated fat, studies have reported decreased consumption [25], increased consumption [26], and no change in consumption [14,24]. Some studies noted increased monounsaturated fat consumption during Ramadan [25,26], while others noted no change [14,24]. However, there appears to be a consensus that polyunsaturated fat consumption does not change during Ramadan [14,24-26]. Also, trans fatty acid consumption was reported to increase during the fast [26].

**Table 1:** Effects of Ramadan fasting on nutrient intake

Outcome	Reference	Subjects	Effect
Energy (kcal)	Adlouni [25]	32 M	↑
	Kassab [51]	44 W	^1^
	Lamine [52]	9 M & 21 W	↑
	Rakicioğlu [27]	21 W	↔^2^
	Al-Hourani [53]	57 W	↔
	El Ati [24]	16 W	↔
	Ibrahim [14]	9 M & 5 W	↔
Carbohydrate (%)	Adlouni [25]	32 M	↑
	Al-Hourani [53]	57 W	↔
	Bouhlel [33]	9 M	↓
	El Ati [24]	16 W	↓
	Maughan [28]	59 M	↓
	Rakicioğlu [27]	21 W	↓
Protein (%)	Adlouni [25]	32 M	↑
	El Ati [24]	16 W	↑
	Maughan [28]	59 M	↑
	Al-Hourani [53]	57 W	↔
	Bouhlel [33]	9 M	↔
	Rakicioğlu [27]	21 W	↔
Fat (%)	Bouhlel [33]	9 M	↑
	El Ati [24]	16 W	↑
	Khaled [26]	276 W	↑
	Rakicioğlu [27]	21 W	↑
	Adlouni [25]	32 M	↔
	Al-Hourani [53]	57 W	↔
	Maughan [28]	59 M	↔

^1 ^Energy intake at day 14 increased compared to pre-fasting values (p < 0.05). Energy intake at day 28 did not change compared to pre-fasting values (p > 0.05).

^2 ^Energy intake increased to an extent that approached significance (p = 0.058).

M = men; W = women

Aside from macronutrients, vitamins and minerals are generally consumed in similar amounts during Ramadan. Examples include: thiamin [27]; niacin [27] folate [27]; vitamin C [14,27]; vitamin E [14,27] calcium [27]; magnesium [27]; potassium [27]; and zinc [27]. Vitamin A consumption was noted to increase during Ramadan [27]. Also, Rakicioğlu and colleagues [27] found that riboflavin consumption decreased to a level that approached significance during Ramadan (p = 0.056).

Mixed findings exist regarding the intake of sodium and fiber during the fast. Regarding sodium, studies reported either decreased consumption [28] or no change in consumption [27]. Regarding fiber, studies noted either decreased consumption [26] or no change in consumption [27].

Collectively, the literature indicates that dietary changes pertaining to kcal intake, as well as macro- and micro-nutrient intake, may or may not differ over the period of Ramadan. These differences, or lack thereof, may be responsible for the discrepancies in health-related outcomes across studies.

Regarding anthropometric variables, body mass index (BMI) may or may not decrease in response to Ramadan fasting. See Table 2 for a presentation of available findings.

**Table 2:** Effects of Ramadan fasting on body mass index

Reference	Subjects	Effect
El Ati [24]	16 W	↔
Ibrahim [14]	9 M & 5 W	↔
Kassab [51]	44 W	↔
Maislos [29]	16 M & 8 W	↔
Maughan [28]	59 M	↔
Rakicioğlu [27]	21 W	↔
Yucel [54]	21 M & 17 W	↔
Al-Hourani [53]	57 W	↓
Bouhlel [33]	9 M	↓
Fakhrzadeh [41]	50 M & 41 W	^1^
Khaled [26]	276 W	↓
Salehi [55]	28 M	↓
Ziaee [31]	41 M & 39 W	↓

^1 ^Body mass index decreased in men (p < 0.05) but did not change in women (p > 0.05).

M = men; W = women

The majority of findings related to total cholesterol, low density lipoprotein cholesterol (LDL-C), and high density lipoprotein cholesterol (HDL-C) are mixed. See Table 3 for a presentation of available findings. Also, heterogeneous findings exist regarding whether Ramadan fasting decreases [29,30] or increases [31] the LDL-C/HDL-C ratio. Finally, the TC/HDL-C ratio appears to decrease during Ramadan [13,29,30,32].

**Table 3:** Effects of Ramadan fasting on blood lipids

Outcome	Reference	Subjects	Effect
Total Cholesterol	Fedail [17]	20 M & 4 W	↑
	Khaled [26]	276 W	↑
	Aksungar [13]	12 M & 12 W	↔
	Bernieh [18]	7 M & 4 W	↔
	El Ati [24]	16 W	↔
	Kassab [51]	44 W	↔
	Ibrahim [14]	9 M & 5 W	↔
	Maislos [29]	16 M & 8 W	↔
	Rakicioğlu [27]	21 W	↔
	Ramadan [34]	13 M	↔
	Sarraf-Zadegan [30]	22 M & 28 W	↔
	Ziaee [31]	41 M & 39 W	↔
	Adlouni [25]	32 M	↓
	Fakhrzadeh [41]	50 M & 41 W	↓
	Salehi [55]	28 M	↓
LDL Cholesterol	Ziaee [31]	41 M & 39 W	↑
	Aksungar [13]	12 M & 12 W	↔
	Maislos [29]	16 M & 8 W	↔
	Sarraf-Zadegan [30]	22 M & 28 W	↔
	Adlouni [25]	32 M	↓
	Fakhrzadeh [41]	50 M & 41 W	↓
HDL Cholesterol	Adlouni [25]	32 M	↑
	Aksungar [13]	12 M & 12 W	↑
	Fakhrzadeh [41]	50 M & 41 W	↑
	Maislos [29]	16 M & 8 W	↑
	Sarraf-Zadegan [30]	22 M & 28 W	↔
	Ziaee [31]	41 M & 39 W	↓

M = men; W = women

Heterogeneous findings have been noted regarding other biochemical outcomes during Ramadan fasting, including blood triglyceride and blood glucose levels. See Table 4 for a presentation of available findings.

**Table 4:** Effects of Ramadan fasting on blood triglycerides and blood glucose

Outcome	Reference	Subjects	Effect
Triglycerides	Kassab [51]	44 W	↔^1^
	Aksungar [13]	12 M & 12 W	↔
	Bernieh [18]	7 M & 4 W	↔
	El Ati [24]	16 W	↔
	Fedail [17]	20 M & 4 W	↔
	Maislos [29]	16 M & 8 W	↔
	Ramadan [34]	13 M	↔
	Salehi [55]	28 M	↔
	Ziaee [31]	41 M & 39 W	↔
	Adlouni [25]	32 M	↓
	Fakhrzadeh [41]	50 M & 41 W	↓
	Ibrahim [14]	9 M & 5 W	↓
Glucose	Bernieh [18]	7 M & 4 W	↔
	Bouhlel [33]	9 M	↔
	El Ati [24]	16 W	↔
	Kassab [51]	44 W	↔
	Maislos [29]	16 M & 8 W	↔
	Maughan [38]	48 M	↔
	Adlouni [25]	32 M	↓
	Ibrahim [14]	9 M & 5 W	↓
	Salehi [55]	28 M	↓
	Ziaee [31]	41 M & 39 W	↓

^1 ^Blood triglycerides increased in obese women to an extent that approached significance (p = 0.05), as cited by authors. Blood triglycerides did not change in lean women (p > 0.05).

M = men; W = women

Little consensus exists regarding blood count outcomes during Ramadan. Hematocrit has been reported to either decrease [28,30], increase [33], or stay the same [34-36]. The majority of studies found that hemoglobin levels do not change [18,35-37], but decreased levels have also been reported [30]. Maughan and colleagues [38] reported that hemoglobin levels decreased in soccer players who were tested in the morning after two weeks of fasting; however, these levels returned to their pre-Ramadan values after two more weeks of fasting. Contrastingly, the same group reported that hemoglobin levels decreased in soccer players who were tested in the afternoon after both two and four weeks of fasting [38]. Serum iron levels have been found to either decrease [34] or remain unchanged [35] during Ramadan fasting. Ramadan and colleagues reported that serum iron levels decreased in sedentary men but did not change in physically active men [34]. Transferrin levels have been reported to increase during Ramadan [38]. Maughan and colleagues reported that serum ferritin levels decreased after two weeks of Ramadan fasting in subjects tested in the morning [38]; however, these levels returned to pre-Ramadan values after two additional weeks of fasting. Contrastingly, the same group noted that serum ferritin levels did not change at any time point in subjects who were tested in the afternoon [38]. Tayebi and coworkers reported that mean corpuscular hemoglobin concentration (MCHC) levels increased in weight-lifters that ceased exercise during Ramadan [36]; in contrast, the same group noted that MCHC levels did not change in weight-lifters that continued to resistance train three sessions per week for 90 minutes per session. Other studies have reported that MCHC levels do not change during the fast [37,39].

Two studies to date have examined the effects of Ramadan fasting on oxidative stress and antioxidant status. Ibrahim and coworkers [14] observed a reduction in malondialdehyde (MDA) concentrations in red cells, while no changes were noted regarding levels of either serum MDA or plasma protein-bound carbonyls. No change was noted in the concentration of glutathione or the activities of glutathione peroxidase and catalase in red cells. Plasma levels of ß-cryptoxanthin and total carotenoids significantly decreased during Ramadan fasting, and plasma levels of vitamin C, β-carotene, lycopene, and lutein were non-significantly reduced. No changes were noted regarding plasma levels of α-tocopherol, γ-tocopherol, retinol, α-carotene, and zeaxanthin. Chaouachi and coworkers [40] reported that subjects experienced an increase in blood levels of vitamin A and a decrease in blood levels of vitamin E during Ramadan.

There are mixed results regarding the effects of Ramadan fasting on blood pressure. Studies have reported that neither resting systolic pressure nor resting diastolic pressure changes during the fast [35,41], but decreases in both variables have also been reported [30]. One study found that systolic (but not diastolic) pressure during steady-state aerobic exercise increased during Ramadan [35].

To summarize, little consensus exists regarding the effects of Ramadan fasting on the majority of health-related outcomes. Many of the discrepancies regarding findings are likely due to differences between studies in daily fasting time, smoking status, medication consumption, and/or dietary norms. Although most manuscripts report daily fasting time, many do not report one or more of the other confounding variables. Future research should be designed so as to eliminate - or minimize the effect of - as many confounding variables as possible. For example, future studies should likely exclude smokers from the subject population so that the effects of daytime smoking cessation are not conflated with the effects of the fast itself. Alternatively, smokers should be allocated to a separate group of fasters, which may then be compared to non-smokers undergoing the fast. The same care should be taken to control for other potential confounding variables, as only through such careful control within the research design will reliable results pertaining to the health effects of Ramadan fasting be obtained.

### Greek Orthodox

There are three principal fasting periods for Greek Orthodox Christians. During the Nativity fast (40 days), fasters abstain from dairy products, eggs, and meat every day. Also, fasters abstain from fish and olive oil on Wednesdays and Fridays during this period. During Lent (48 days), fasters abstain from dairy products, eggs, and meat every day. Additionally, fasters abstain from olive oil on weekdays during this period and abstain from fish every day except for March 25^th ^and Palm Sunday. During the Assumption (15 days), fasters abstain from dairy products, eggs, and meat. Also, fasters abstain from olive oil on weekdays during this period and abstain from fish every day except for August 6^th^. In addition to these principal fasts, every Wednesday and Friday that falls outside of a principal fasting period calls for the proscription of cheese, eggs, fish, meat, milk, and olive oil. Exceptions to these proscriptions occur on the week following Christmas, Easter, and the Pentecost. Collectively, dietary consumption is restricted for 180 - 200 days each year. The Greek Orthodox Christian diet consists largely of bread, fruits, legumes, nuts, seafood, snails, and vegetables during fasting periods [42]. This diet can be viewed as a variant of vegetarianism and also as a form of DR.

Daily kcal intake may or may not decrease during the fasting periods [42-46]. In terms of percentage of energy consumption, there appears to be a consensus that Greek Orthodox Christian fasting increases carbohydrate intake and decreases fat intake [42-44,46]. Also, the amount of protein intake relative to carbohydrate and fat intake may or may not decrease during Greek Orthodox Christian fasting [46]. When expressed as an absolute amount, both total fat and total protein intake decrease during the fasting periods, while total carbohydrate intake does not change [43]. Both saturated fat and trans-fatty acid consumption decrease during fasting periods, and monounsaturated fat consumption does not change [43,44,46]. Additionally, polyunsaturated fat consumption may or may not decrease during Greek Orthodox Christian fasting [43,44,46].

In terms of vitamin and mineral intake, both riboflavin [43] and calcium [43,44,46,47] intake decrease during fasting periods. On the other hand, magnesium intake increases during these periods [43,47]. The intake of the following vitamins and minerals do not appear to change during fasting periods: vitamin A [43,44,46]; thiamin [43]; niacin [44]; vitamin C [43-45] (Sarri, 2009); vitamin E [43,44,46]; phosphorus [43]; potassium [43,47]; and zinc [43]. Some studies found that folate consumption increases during Greek Orthodox Christian fasting [43,44], while another study noted no difference in consumption between fasters and non-fasters [47]. Sodium intake has been found to either decrease [47] or not change [43] during the fasts. Also, vitamin B_12 _may or may not decrease during Greek Orthodox Christian fasting [43,44,46].

In terms of anthropometric outcomes, BMI may or may not decrease during fasting periods [42,46]. Also, the average body mass of Greek Orthodox Christian monks was observed to decrease during a fasting week by an amount that approached significance (p = 0.059) [43].

Regarding biochemical outcomes, both total cholesterol and LDL-C levels decrease during fasting periods [42,43,46]. One study reported a decrease in HDL-C levels [42], while other studies reported no change [43,46]. The LDL-C/HDL-C ratio does not change during Greek Orthodox Christian fasting [42]. One study noted a decrease in TC/HDL-C ratio [43], while another study noted no change [42]. Regarding triglyceride levels, one study found an increase during fasting periods [43], while other studies noted no change [42,46]. Besides blood lipids, Greek Orthodox Christian fasting may or may not decrease blood glucose levels [42,43,46]. Also, several studies have reported that fiber intake increases during fasting periods [42-45,47], although one study reported no change [46].

Sarri and coworkers compared other hematological changes in fasters and non-fasters during the Christmas fasting period [45]: the authors reported a relative increase in serum ferritin levels, a relative decrease in MCHC levels, and no relative change in levels of hemoglobin, serum iron, and transferrin. The authors also reported that the non-fasters' hematocrit values significantly declined compared to the fasters' hematocrit values. Despite this decrease, the non-fasters' hematocrit end values were actually higher than the fasters' hematocrit end values, and both end values were within normal ranges.

There are conflicting findings on the effects of Greek Orthodox Christian fasting on blood pressure. One study found that systolic blood pressure increased during fasting periods [43], while another study found no change in blood pressure when fasters were compared with non-fasters [47]. One study reported that non-fasters' diastolic blood pressure decreased significantly during fasting periods when compared to the changes in fasters' diastolic blood pressure [47], while another study reported that fasters' diastolic blood pressure did not change during fasting periods [43].

Although no study to date has examined oxidative stress in response to Greek Orthodox Christianity fasting, a recent study measured serum antioxidant levels before and after the Nativity fast [46]. This investigation found that serum retinol, the retinol:TC ratio, serum α-tocopherol, and the α-tocopherol:TC ratio each decreased following this fast.

In sum, Greek Orthodox Christian fasting appears to lower body mass. Carbohydrate intake appears to increase, while the intake of protein, total fat, saturated fat, and trans fatty acids decrease during fasting periods. Both total and LDL-C decrease, although the LDL-C/HDL-C ratio does not appear to change. Fiber intake increases during fasting periods, which may partly explain the change in serum lipids. The intake of most vitamins and minerals does not appear to change during these periods, although riboflavin and calcium intake each appear to decrease, and magnesium intake appears to increase. Also, one investigation reported increases in serum retinol and serum α-tocopherol. More research remains to be performed on hematological variables and blood pressure during fasting periods due to both the lack of previous research and the mixed findings. Moreover, given that each fasting period has unique durations and dietary precepts, future research should examine these fasting periods both separately and collectively.

### Daniel Fast

A popular fast practiced by Christians, the Daniel Fast derives from the Biblical story of Daniel (1:8-14 NIV), in which Daniel resolved not to defile himself with the royal food and wine and requested permission to consume nothing but vegetables (pulse) and water for 10 days. Later in the same book (Daniel 10:2-3 NIV), Daniel again followed a 21 day period of fasting, during which time he ate no choice food (meat or wine). Based on these two passages, a modern day Daniel Fast involves *ad libitum *intake of specific foods, but the food choices are restricted to fruits, vegetables, whole grains, legumes, nuts, seeds, and oil. This plan resembles a vegan diet, which has been reported to yield health-enhancing properties [48,49].

Like Greek Orthodox Christian fasting, the Daniel Fast may be viewed as a form of DR. However, unlike the Greek Orthodox Christian fasts, or a simple vegetarian diet, a Daniel Fast is much more stringent; this is because refined foods, white flour, preservatives, additives, sweeteners, flavorings, caffeine, and alcohol are each forbidden. Nonetheless, because individuals traditionally follow the Daniel Fast for strict religious purposes in an attempt to become closer to God during a time of extended prayer, anecdotal reports and preliminary scientific study have indicated excellent compliance. The Daniel Fast is most commonly partaken for 21 days, although longer (e.g., 40-day) and shorter (e.g., 10-day) fasts have been observed. While the Daniel Fast can be partaken at any time of the year, it is often practiced during the month of January in order to begin the New Year through fasting and prayer.

To test the health benefits of the Daniel Fast within a laboratory-based protocol, we recently enrolled men and women of varying age (20-62 yrs) into a 21-day Daniel Fast [50]. Pre and post intervention, subjects underwent a variety of laboratory tests including measures of body weight and body composition (measured via dual energy x-ray absorptiometry), resting blood pressure and heart rate, fasting blood measures of oxidative stress, inflammation, blood lipids, insulin, and glucose. Subjects also rated their compliance to the fast, as well as their overall mood and satiety during the 21-day fasting period.

We noted excellent compliance to the fast (> 98%), as well as both excellent results for overall mood and satiety (7.9 ± 0.2 using a 10 point scale). As expected, a reduction was noted in the following variables from the seven days prior to starting the fast to the final seven days of the fast: total kcal, protein, total fat, saturated fat, trans fat, and cholesterol. Also, an increase was noted in the following: carbohydrate, fiber, and vitamin C.

The following variables related to cardiovascular disease risk were significantly lower following the fast as compared to before the fast: total cholesterol, LDL-C, SBP, and DBP. Insulin, HOMA-IR, and C-reactive protein were all lowered to a clinically meaningful extent, but this decrease failed to reach statistical significance. Due to the drastic decrease in total cholesterol (~19%), HDL-C was lower after the fast as compared to before the fast (55.65 ± 2.50 vs. 47.58 ± 2.19 mg·dL^-1^). Although body weight and body fat were reduced slightly, no significant difference was noted. In regards to measures of blood antioxidant status and oxidative stress, a significant increase was noted in Trolox Equivalent Antioxidant Capacity and nitrate/nitrite (a surrogate marker of nitric oxide), while a significant decrease was noted in MDA (manuscript in progress).

The above findings demonstrate that a 21-day Daniel Fast can significantly improve several markers of overall health, particularly those related to cardiovascular and metabolic disease. Larger scale, randomized studies are needed to extend these preliminary findings. Future studies should possibly consider extending the duration of the fast, as well as modifying food choices in an effort to maintain or improve HDL-C levels.

## Overall Summary and Conclusions

Comparisons between different studies of Ramadan fasting are difficult due to several confounding variables. Daily fasting time is highly dependent upon the time of the seasonal year that Ramadan occurs as well as the location's latitudinal distance from the equator. The percentage of subjects who smoke, consume oral medications, and/or receive intravenous fluids can greatly affect a study's findings, because these activities are forbidden during the daylight hours of Ramadan. Finally, food choices and eating habits can vary greatly between different cultures. Collectively, these confounding variables likely explain the lack of consensus regarding the effects of Ramadan fasting on the majority of health-related biomarkers observed in this review. To the extent that it is possible, future work should attempt to minimize the effect that these confounding variables have on findings.

During the Greek Orthodox Christian fasting periods, the daily intakes of protein, fat, saturated fat, trans-fatty acids, riboflavin, and calcium are reduced. Also, these fasts are associated with lower levels of the following: body mass, total cholesterol, LDL-C, and the LDL-C/HDL-C ratio. Intake of fiber and carbohydrate (expressed as a percentage of macronutrient intake) increase, while no changes occur regarding the intake of monounsaturated fat as well as most vitamins and minerals. To date, only a few investigations have examined the health-related effects of these fasting periods. Clearly, more work remains to be performed, particularly regarding hematological and blood pressure variables. Also, each principal fasting period should be studied both separately and aggregately due to their differences in food proscriptions and durations.

Only one study to date has examined the health-related effects of a Daniel Fast. This study reported meaningful improvements in blood pressure, blood lipids, and biomarkers of oxidative stress. Moreover, insulin, HOMA-IR, and C-reactive protein were all lowered to a clinically meaningful extent. However, the study also noted an undesirable lowering of HDL-C. Future work should modify food choices in an effort to maintain HDL-C. Also, future research should examine the tolerability and health-related effects of partaking in a Daniel Fast for longer durations.

Collectively, the religious fasts featured in this review emphasize the importance of *quality *dietary intake in eliciting favorable effects on health. Both the Daniel Fast and the Greek Orthodox Christian fasts are associated with many favorable health outcomes despite the fact that fasters can eat as much as they desire. On the other hand, whether or not Ramadan fasting elicits favorable health outcomes appears to greatly depend on the food choices of the fasters. This is not to suggest that the *quantity *of food intake has no bearing on health; hundreds of studies have demonstrated the virtue of restricting kcal consumption [1]. Rather, these religious fasts reaffirm the position that one can improve his or her diet without being compelled to reduce food intake.

Diet plays an integral role in the religious customs of a variety of faiths. For many religions, this role is manifested in the form of specialized fasting periods. Although religious fasting is often a time of great spiritual growth, it can also be a time of great improvement to one's physical health. This review highlights the potential of different religious fasts as forms of dietary modification. It is our hope that the information provided within this review will initiate the design and performance of future investigations focused on the health benefits of religious fasting.

## Competing interests

The authors declare that they have no competing interests relative to the material presented within this article.

## Authors' contributions

JFT was chiefly responsible for reviewing the relevant literature and writing this article. RJB provided the article outline and assisted with the writing and editing of this article. Both authors read and approved the final manuscript.
